# Groundwater quality assessment using water quality index and principal component analysis in the Achnera block, Agra district, Uttar Pradesh, Northern India

**DOI:** 10.1038/s41598-024-56056-8

**Published:** 2024-03-05

**Authors:** Shahjad Ali, Sitaram Verma, Manish Baboo Agarwal, Raisul Islam, Manu Mehrotra, Rajesh Kumar Deolia, Jitendra Kumar, Shailendra Singh, Ali Akbar Mohammadi, Deep Raj, Manoj Kumar Gupta, Phuyen Dang, Mehdi Fattahi

**Affiliations:** 1 Department of Applied Sciences, Anand Engineering College, Agra, Uttar Pradesh India; 2 Department of Environmental Science and Engineering, IIT(ISM), Dhanbad, Jharkhand India; 3 Department of Civil Engineering, GLA University, Mathura, India; 4 Department of Applied Science (Mathematics), G.L. Bajaj Group of Institutions, Mathura, India; 5 Department of Mathematics and Computing, Madhav Institute of Technology and Science, Gwalior, India; 6 Department of Mechanical Engineering, Anand Engineering College, Agra, India; 7 Department of Environmental Health Engineering, Neyshabur University of Medical Sciences, Neyshabur, Iran; 8 Department of Environment Science and Engineering, SRM University-AP, Amaravati, Andhra Pradesh India; 9 Department of Applied Science, Bundelkhand Institute of Engineering and Technology (BIET), Jhansi, India; 10 Institute of Research and Development, Duy Tan University, Da Nang, Vietnam; 11 School of Engineering and Technology, Duy Tan University, Da Nang, Vietnam

**Keywords:** Water quality index, Schollar diagram, Hydrochemistry, Principal component analysis, Environmental chemistry, Environmental monitoring

## Abstract

The qualitative and quantitative assessment of groundwater is one of the important aspects for determining the suitability of potable water. Therefore, the present study has been performed to evaluate the groundwater quality for Achhnera block in the city of Taj, Agra, India, where groundwater is an important water resource. The groundwater samples, 50 in number were collected and analyzed for major ions along with some important trace element. This study has further investigated for the applicability of groundwater quality index (GWQI), and the principal component analysis (PCA) to mark out the major geochemical solutes responsible for origin and release of geochemical solutes into the groundwater. The results confirm that, majority of the collected groundwater samples were alkaline in nature. The variation of concentration of anions in collected groundwater samples were varied in the sequence as, HCO^3−^ > Cl^−^ > SO4^2−^ > F^−^ while in contrast the sequence of cations in the groundwater as Na > Ca > Mg > K. The Piper diagram demonstrated the major hydro chemical facies which were found in groundwater (sodium bicarbonate or calcium chloride type). The plot of Schoellar diagram reconfirmed that the major cations were Na^+^ and Ca^2+^ ions, while in contrast; major anions were bicarbonates and chloride. The results showed water quality index mostly ranged between 105 and 185, hence, the study area fell in the category of unsuitable for drinking purpose category. The PCA showed pH, Na^+^, Ca^2+^, HCO^3−^ and fluoride with strong loading, which pointed out geogenic source of fluoride contamination. Therefore, it was inferred that the groundwater of the contaminated areas must be treated and made potable before consumption. The outcomes of the present study will be helpful for the regulatory boards and policymaker for defining the actual impact and remediation goal.

## Introduction

Quality of life is associated with quality of water we consume. Out of all water resource, groundwater is one of the important drinking water resources^1,2^. In the arid and semi-arid regions, especially for the developing countries like India and Bangladesh, the rapid population growth associated with intensive developmental activities results in a severe increase in water demand^1–3^. The day-to-day degradation of groundwater quality has now become one of the serious challenges in the world. Billions of people across the globe are compelled to consume the polluted water due to the scarcity of potable water, and therefore the scarcity of groundwater is an alarming threat to the humans. It has now been well established that groundwater is at higher risk, in terms of its purity^3–6^. In remote areas, the situation of groundwater is even more miserable, due to over withdrawal of groundwater. The residents of urban areas have to walk several kilometers to fetch potable water^5,6^. The government and various non-governmental organizations (NGOs) are working hard enough to provide contaminants free potable water to every individual^7^. It has been reported in previous literatures that, contaminants like heavy metals, pesticides, organic and inorganic pollutant are causing the serious human health disease such as hypertension, hypocalcaemia, kidney stones, gastro-renal discomfort, arterial calcification, thrombosis^8–12^. Apart from the availability of heavy metals in drinking water, the presence of nitrogen has also been proven as the strong potential threat to the quality of drinking water^13–15^. With the increasing groundwater pollution, it is essential to analyze groundwater chemical characteristics and evaluate groundwater quality for water supply purpose. In this regard, methods like groundwater quality index (GWQI), the fuzzy comprehensive method and the health risk weight method (HRWM) have been widely used by researchers. Among these methods, the water quality index (WQI) has been more commonly used by international researcher due to its simple calculation, practicality, and versatile applications^16–18^.

GWQI is a mathematical expression that can be used to determine the quality of groundwater in different locations globally. The idea of GWQI has been kept to assess the water quality throughout diverse world-wide areas^19,20^. It is an important tool for the decision-makers to choose the best method for pre-remediation goal^21–24^. As a result, it has become a crucial component in the evaluation of water quality. In India, multiples research in different areas indicated that the sources of drinking water contain heavy metals like cadmium, lead, mercury, arsenic, and manganese. Also, research findings have shown elevated levels of fluoride, which exceed World Health Organization (WHO), limits of 1.5 ppm^25–31^. In a study on the Ramganga aquifer of Bareilly District in Uttar Pradesh (India), it was found that the groundwater which were extracted from shallow aquifer contain high percentage of zinc and nickel, whereas the samples collected from deep aquifer consists of heavy metals like copper, cobalt, nickel, manganese, cadmium, and zinc^32^. Previous research revealed that the quality of drinkable water in several regions of northern India is unfit for drinking. About 35 districts are reported to have been found variously affected with arsenic toxicity^31–33^.

Considering the above mention highlights, this study was undertaken to achieve the following objectives: (a) the primary aim of the present research work was to explore the level of contamination in one of the unexplored parts of northern India, which has not been marked before in the previous studies, i.e., the city of Taj Mahal, Agra, India, (b) Further, qualitative of groundwater has been estimated by hydro-chemical analysis and GWQI estimation respectively, (c) to use PCA for the determination of the components that influence the discharge of hydro chemical solutes into the groundwater, (d) to investigate the correlation between hydro chemical parameters and their common source of origin.

## Materials and methods

### Study area and its geology

**Figure 1:**
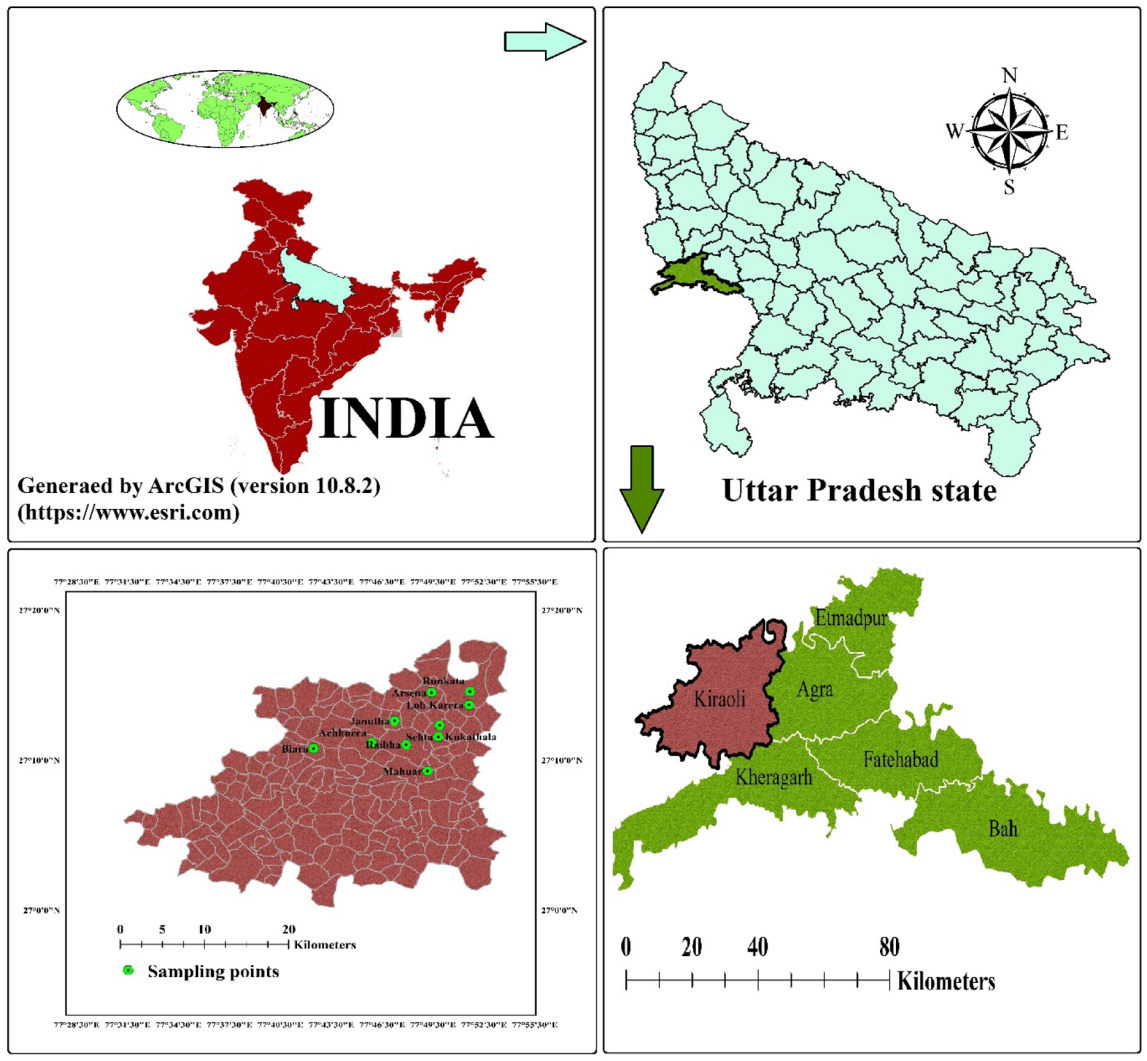
Locations of Achhnera block in Agra, North India.

The present study was focused on one of the blocks of city of Taj-Mahal, Agra, India. This city is located on the bank of river Yamuna, Uttar Pradesh, India, between 27°11′ N and 78°02’ E (Fig. 1). With a rapid pace of population increase, it is one of biggest city in northern India. There are 15 administrative blocks, 904 villages, and six tehsils in the Agra district. With reference to the 2011 India’s Census, the Agra district has over 7 million households, with a population of 44, 18,797 of which 53.52% are males and 46.48% are females. The weather of the sampled area was semi-arid to sub-tropic type, with an average annual precipitation of 687 mm and evaporation of 1466 mm/year. The average temperature range varies from21.9 to 45 °C in hot days and 3.9–32.2 °C in cold days. Annually, the rainfall averages to about 687.2 mm due to the southwest monsoon, and consequently the daily relative humidity ranges from 30 to 100%^34^.

The study area occupies a part of Indo-Gangetic plain and its major part is underlined by alluvial sediments of quaternary age encompasses primarily a sequence of clay, silt, sand of different grades, gravels and kankar in different magnitude. In this study region, over 90% of the population use groundwater for drinking purposes thus, investigations of quality of groundwater are among the highest priorities. In this study, ArcGIS (version 10.8.2) was used for geographical data processing and visualization. ArcGIS is a product of Esri and more details about the software can be found on their official website (https://www.esri.com).

### Sample collection and hydro-geochemical analysis

The samples were collected from the selected area via tube wells, hand pumps and wells. All samples were collected in a time interval of one year from February 2022 to January 2023. A total of 50 groundwater samples from 10 villages (5 × 10) were collected and preserved in polypropylene bottles at 4ºC. The sampling locations have been plotted through ArcGIS 10.2 (Fig. 1). To stabilize the pH, conductivity and temperature of the sampling area, the hand pumps were used for some time before collection of samples. All chemical used were of analytical grade (Merck Darmstadt, Germany). During the analysis of samples standard methods were used as given in APHA 2012. Concentration of fluoride (F^−^), sulphate (SO_4_^2−^) and nitrate (NO^3−^) ions were determined by using spectrophotometer. Mohr’s method (AgNO_3_) was used to determine chloride (Cl^−^) content in the samples. Titration and flame photometry method was used to determine hardness, alkalinity, Mg^2+^, Ca^2+^, Na^+^, and K^+^ ions in the water samples. Total dissolved solids (TDS) and pH were analyzed by multi-parameter kit^35^. The results were counter checked by the calculation of cation and anion balance. The estimated error was less than ± 5% for all the collected samples.

#### Calculation of the WQI of the samples

The WQI model is an interested tool for assessing groundwater and surface water quality. It uses aggregation techniques that allow conversion of extensive water quality data into a single value or index. Globally, the WQI model has been applied to evaluate water quality according to local criteria. The guidelines laid by WHO for drinking water are illustrated in (Table 1).

**Table 1:** Guidelines for potable water quality^38,39^.

Variables (mg/L)*	BIS, 10500 standards	WHO standards
F^−^	1–1.5	1.5
TA	200–600	500
TDS	500–2000	1000
Cl^−^	250–1000	250
Na^+^	–	200
K^+^	–	12
SO_4_^2–^	200–400	250
NO^3–^	45	50
Mg^2+^	30–100	50
Ca^2+^	75–200	200
TH	200–600	500
pH	6.5–8.5	8.5

*TH* total hardness; *TA* total alkalinity.

*pH is unitless.

#### Calculation of the unit weight (Wn)

The following equations refer to the calculation Wn (Eq. 1).

1 $${\text{Wn}} = {\text{K}}/{\text{Xs}}$$

where $K = \frac{1}{{\sum {Xs} }}$, Wn: unit weight parameter^36,37^; Xs: suggested standard for parameter, K: Proportionality Constant; n = number of different water quality parameters.

#### Calculation of groundwater quality rating

Quality rating scale Qn was computed according to WHO guidelines using the relation of Eq. (2).

2 $$Qn = 100 \times \frac{Xn - Xi}{{Xs - Xi}}$$

Xn—actual concentration of water quality parameters; Xi—ideal value of different water quality parameters (0 for all parameters except pH 7 ppm).

#### Estimation of Water Quality Index (WQI)

3 $$WQI = \frac{{\sum {QnWn} }}{{\sum {Wn} }}$$

Various researchers have followed the above method to calculate WQI. Generally, the water quality index (WQI) differentiates potable water into different classes, as shown below in Table 2^36,38–40^.

**Table 2:** Categorization of drinking water by ranges of WQI.

WQI range	Water quality
> 100	Unfit water
76–100	Poor water
51–75	Moderately poor
26–50	Palatable water
0–25	Potable water

#### Principal component analysis (PCA)

A well-reported statistical approach in ground water research, the principal component analysis. The data clarification is obtained along the hidden factor created by original factors such as water quality indicators by regard at the key source of variance in the data. The hidden parameters from a matrix composed of factor loading (weight of principal variable) and factor score (prediction of sampling location on the principal component axis). The PCA has been executed in this study to determine homologous behavior and common origin of different physicochemical properties of groundwater. Further PCA was carried out to identify the various factors responsible for release of contaminants into groundwater^41,42^.

### Statistics used and Data analysis

The data was analyzed using SPSS 16.0 (SPSS Inc. Chicago, USA) and Microsoft Excel 2013. Through SPSS 16.0 spearman correlation was calculated to know the inter-relationship between various hydro-chemical solutes^25^. Further Spatial distribution map has been drawn using arc GIS-10.2 by ESR to evaluate the spatial distribution of fluoride from samples collected from different parts of villages.

## Result and discussion

### Hydrochemistry of groundwater of Achhnera block, Agra

**Figure 2:**
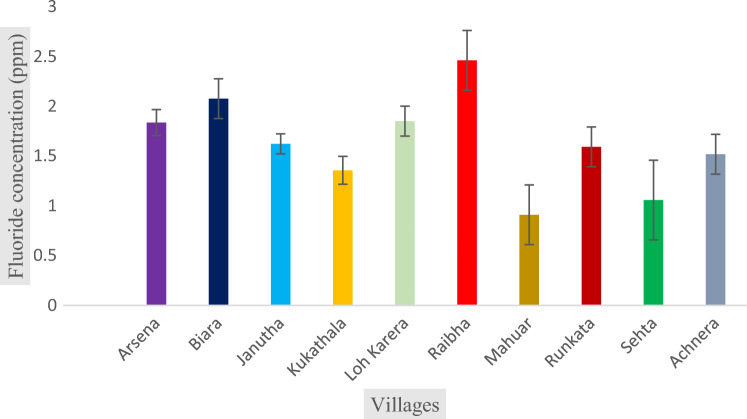
Fluoride concentration in Achhnera block.

The physiochemical properties of groundwater samples have been presented in Table 3. The alkalinity of the groundwater sample has been found in the range from 187 to 493.8 ppm, with an average value of 343 ppm, which is within the permissible limit of 600 ppm^39^. TDS of the samples was very high from 801 to 2065 ppm with the average value of 1327 ppm, which is higher than prescribed limit^39^. The concentration of chloride ranged from 226 to 814 ppm with the average value of 470 ppm. The concentration of sodium, potassium, sulphate, and nitrate ions were found in the range of 165–680 ppm, 12–67 ppm, 37–114 ppm, and 4.6–11 ppm, respectively. The hardness was observed between the ranges of 155 to 485 ppm with an average value of 320 ppm, and correspondingly, the concentration of calcium and magnesium ions was ranged from 64–160 ppm to 6.8–32 ppm, respectively. The most prominent anion found in underground water samples was HCO^3−^, but some samples had Cl^−^ ions as the most prominent anion. Out of all the samples collected, about 50% of them have pH values above the permissible limit of WHO and BIS standards (IS: 10500, 2012) i.e., 6.5–8.5^38,39^. The concentration of fluoride in the sampled water was found in the range of 0.910 to 2.46 ppm, with the average value of 1.628 ppm as shown as in Fig. 2. The result demonstrated that the concentration of fluoride ion was on elevated side, crossing the permissible limit of WHO (1.5 ppm)^38^.

**Table 3:** Physiochemical properties of groundwater samples at Achhnera block.

Parameters (mg/L)	Minimum	Maximum	Mean	SD
F^−^	0.910	2.460	1.628	0.46
TA	187.00	493.80	326.280	98.47
TD	801.20	2065.60	1327.360	391.85
Cl^−^	226.20	814.20	446.540	186.95
Na^+^	165.00	680.00	343.400	152.81
K^+^	12.200	67.200	29.080	15.218
SO_4_^2–^	37.200	114.20	60.340	22.387
NO^3–^	4.600	11.000	7.020	2.304
Mg^2+^	6.840	32.600	13.464	8.137
Ca^2+^	64.000	160.800	82.420	28.59
TH	155.80	485.800	241.700	100.32
pH	8.008	8.960	8.499	0.29

In Korea area, Kim et al. studied the co-contamination of arsenic and fluoride in the groundwater of an alluvial aquifer and reported that the concentrations of fluoride ions among the total 50 samples collected, 35 samples have increased level, which indicated that the soil and the rock of that region contain fluoride-rich minerals^43^. In a similar kind of study carried out by Ali et al.2021, also showed the elevated groundwater fluoride in some blocks of the Agra district and it was observed that the concentration of fluoride in the range of0.14 to 4.88 mg/L^3^. Another study carried out by Ansari and Umar (2019), found very much similar results in Unnao, Uttar Pradesh (India), and the concentration of fluoride was reported in the range between 0.06 to 1.83^44^. A very similar study performed by Chaurisiaya et al. (2018) observed the concentration of fluoridebetween0.28 to 2.01 in Varanasi, Uttar Pradesh, India. Similarly, in some other previous research, the concentration of fluoride ions was ranged from 0.32 to 3.5 in Banda, Uttar Pradesh^45^. Tiwari et al. (2016) reported the elevated range of fluoride concentration i.e., between 0.41 and 3.99 in Pratapgarh, Uttar Pradesh, India^46^. Dev and Raju (2014) found the fluoride concentration between0.08 to 6.7 in Sonbadra, Uttar Pradesh^47^. Hence, it may be inferred that the major portion of northern India is endemic to elevated fluoride concentration (Table 4).

**Table 4:** Distribution table of fluoride content in groundwater in Uttar Pradesh, North India.

Area (India)	Concentration of fluoride (ppm)	Sources of fluoride ions	References
Agra	0.91–2.67	Geogenic and rocks of fluoride bearing minerals in groundwater	Present work
Agra	0.14–4.88	Found in rocks bearing fluoride minerals which interact with water	^57^
Unnao	0.06–1.83	Due to agricultural activity and Brick ash	^44^
Varanasi	0.28–2.01	Geogenic	^45^
Banda	0.32–3.5	From fluoride minerals	^46^
Sonbhadra	0.08–6.7	Occurring due to natural causes mainly due to rock-water interaction	^47^

### Geochemical characterization of Achhnera block

**Figure 3:**
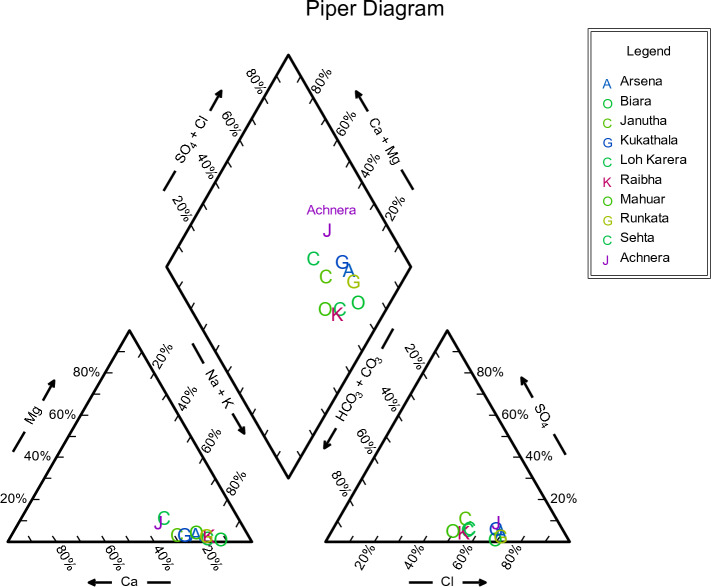
Piper plots for groundwater samples at Achhnera block, Agra, North India.

**Figure 4:**
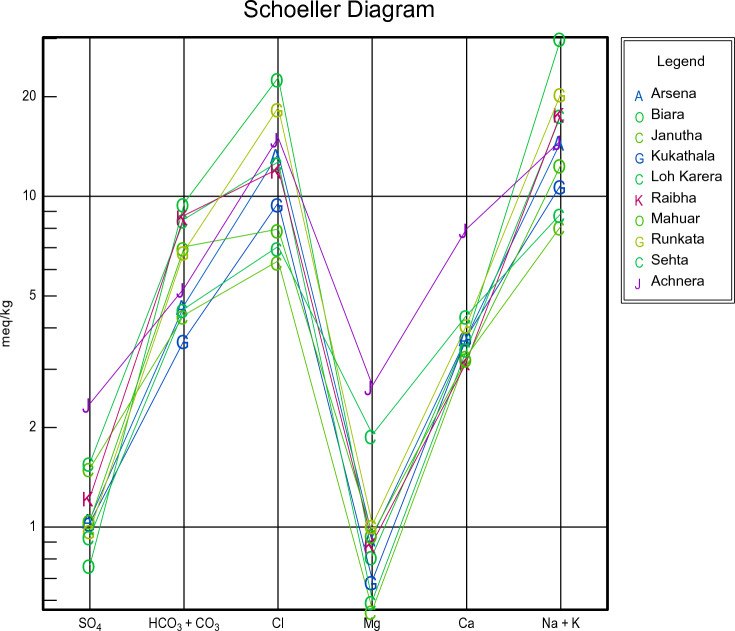
Schoeller diagram Achhnera block, Agra, North India.

For all groundwater samples, the primary dissolved ions were shown through Piper trilinear diagrams and Schoellar diagrams to comprehend the geochemical progression of groundwater. AqQa v1.X, a Rock ware program, was used to plot the diagrams. Separate ternary plots revealed the cations and anions in the piper diagram. Magnesium, calcium, and sodium, potassium was the apex of cation plot while chloride, sulphate, and carbonate, and bicarbonate ions were the apexes of anions plot (Fig. 3). The predominant cation present in the samples was sodium. As a result, the water quality of Achhnera region was classified as either Na^+^/HCO^3−^ or Na^+^/Cl^−^ type, and Ca^2+/^HCO^3−^ type. When fluorite get dissolves in water containing sodium bicarbonate, there is often a moderate correlation between increased fluoride levels due to the presence of bicarbonates^48^. Ionic components of groundwater samples have been displayed in Schoellar diagram (Fig. 4). The primary ionic components of groundwater are SO_4_^2−^, HCO^3−^, Cl^−^, Mg^2+^, Ca^2+^, Na^+^, and K^+^, and their concentrations are shown in the semi-logarithmic Schoellar diagram as equivalents per million per kilogram of solution (meq/kg). Each ion's concentration in each sample was shown by points on six evenly spaced lines, and those points were linked by a line.

In one of the studies on Poyang Lake, China, the presence of nitrogen-nitrate was reported as major threat to the lake with the extensive ongoing agricultural practices^49,50^. In the study, the multi-methods which include grey correlation analysis, Pearson correlation, mathematical statistics, and human health risk assessment were used for the investigation of spatiotemporal variations and potential risks of nitrogen.

### Principal component analysis (PCA)

The PCA has been executed in this study to determine homologous behavior and common origin of different physicochemical properties of groundwater. The values of different Principal Components (PCs) can be considered under strong, moderate, and weak loadings, if their value ranges from 1–0.75, 0.75–0.50 to 0.50–0.30, respectively. The application of PCA in the present study is to obtain correlations between the hydro-chemical components of the groundwater samples.

**Figure 5:**
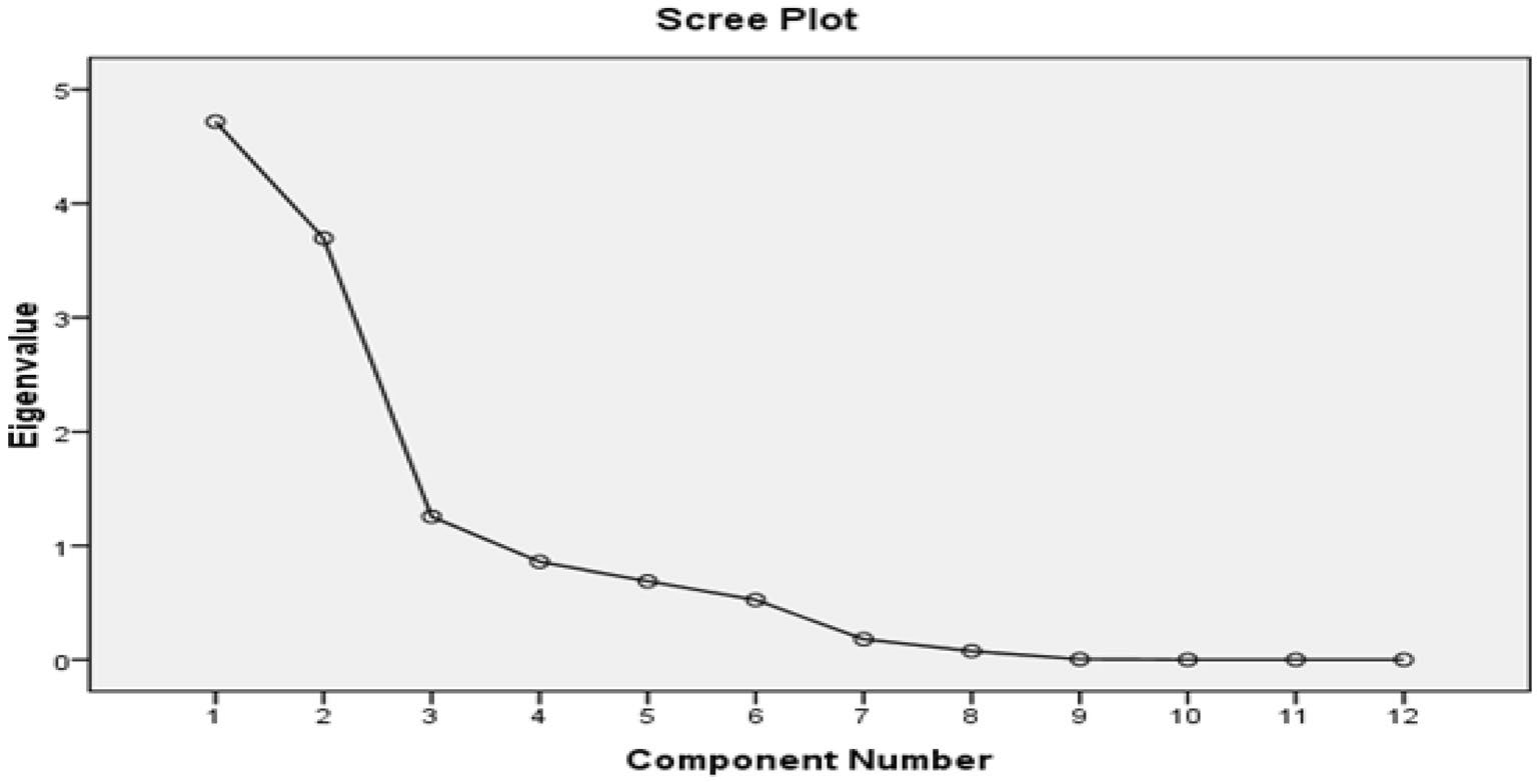
Scree plot of PCA of Achhnera block, Agra, Northern India.

**Figure 6:**
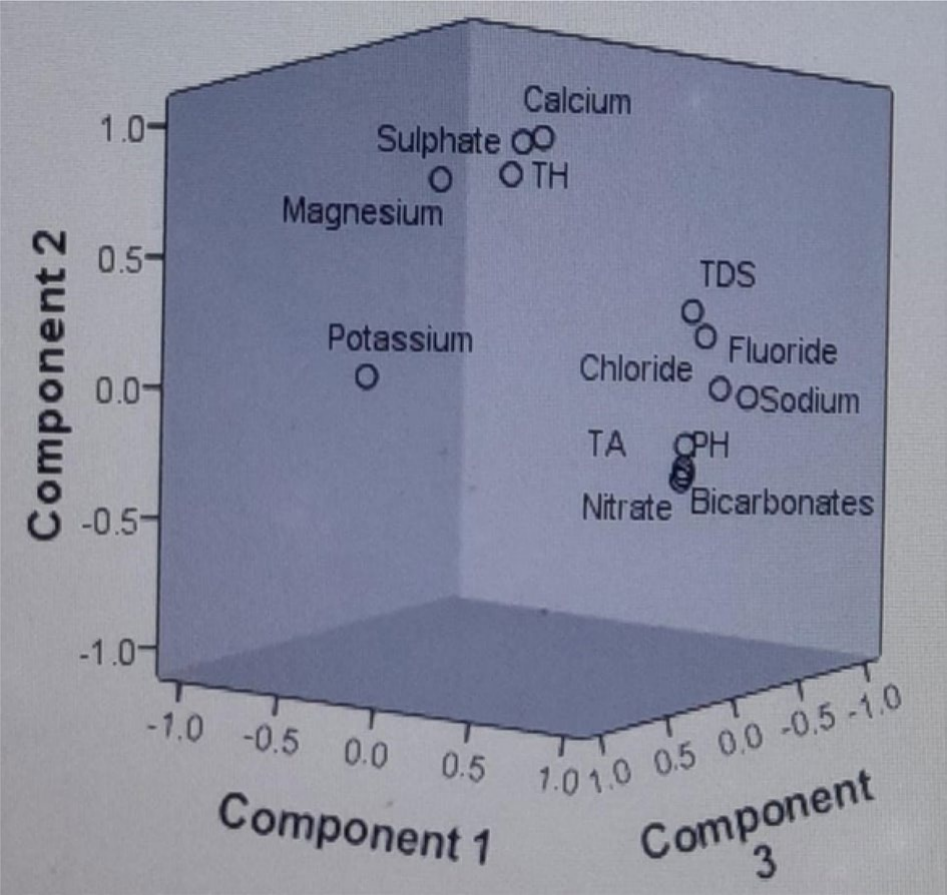
Component plot in rotated space of physico-chemical components of Achhnera block, Agra, North India.

The PCA of the groundwater samples revealed that the variables are inter-correlated with 38.29% of the total variance. As per Kaiser Criterion, the PCs values, whose eigen values were found more than one, can be considered in factor analysis^51^. After varimax rotation, only three PCs values were found more than one, as shown in the scree plot Fig. 5, and the rest can be ignored as their eigen values have been found less than one. Hence, three principal components have been extracted for the consideration. Table 5 showed the variance in the three PC values which is 38%, 32% and 10.85% reasonable correspondingly; hence, the rest of the components can be ignored. Principal component one (PC-1) comprised TDS, NO^3−^, HCO^3−^, Na^+^, TA, and fluoride with moderate to strong loading. Fluoride ion in PCA-1 showing the moderate to strong loading with TDS, NO^3−^, HCO^3−^, Na^+^ and Ca^2+^, which appeared to be linked with geological origin fluoride in the present block, and their origin has been significantly correlated. The changes in the concentration of fluoride were directly related with the TDS, NO^3−^, HCO^3−^, Na^+^, Total Alkalinity, which can be explained due to the evolution of the fluoride from the fluoride bearing minerals present in host rocks and their interaction with groundwater. Therefore, it is concluded that there are no human sources of fluoride in groundwater, indicating that it is obtained geologically. Principal component two (PC-2) includes TH, Ca^2+^ and Mg^2+^ showing high positive factor loadings while in case of principal component three (PC-3) includes pH, Cl^−^, moderate to weak loading. Thus, it can be predicted from the PCA that the component one represents the controlling factors, which is responsible for rerelease of fluoride ions, as all sensitive parameters (TDS, NO^3−^, HCO^3−^, Na^+^ and Ca^2+^) of groundwater have moderate to strong loading with respect to all other principal components (Fig. 6). While other sensitive parameters (TDS, NO^3−^, HCO^3−^, Na^+^ and Ca^2+^) of groundwater show moderate to strong loading with respect to all other principal components.

**Table 5:** Principal component analysis of groundwater samples in Agra region, Northern India.

Parameter (ppm)	Component		
	1	2	3
Component matrix^a^			
F¯	.823	.186	− .143
TA	.853	.125	.265
TDS	.740	.614	.122
Cl¯	.792	.492	.052
Na^+^	.938	.170	− .189
K^+^	− .435	.213	.810
HCO^3−^	.840	− .004	.210
NO3–	.780	− .129	.033
Mg^2+^	− .440	.833	.256
Ca^2+^	− .158	.943	− .150
TH	− .220	.933	− .076
pH (unitless)	.764	− .165	.015

### Correlation analysis of Achhnera block

The correlation coefficient data of rural parts of Achhnera block, Agra region, North India are tabulated in Table 6. Alkalinity was found to increase due to the replacement of fluoride with hydroxide ions. A positive correlation of concentration of hydrogen ion and sodium ion is observed with fluoride ion. The region may be due to high pH. Many researchers have found in their experimental work that there is a strong correlation ship between F^−^ and H^+^ ions as they have a strong tendency of combining and forming HF^52–54^. The concentration of sodium and bicarbonates have shown positive correlation with fluoride, which can be explained due to the high alkalinity in the sampled water, resulting in the dissolution of fluoride in groundwater^51–54^.

**Table 6:** Correlation coefficient of water quality parameters.

Parameters	pH	TDS	TA	TH	Ca^2+^	Mg^2+^	Na^+^	K^+^	SO_4_^2−^	Cl^−^	NO_3_^−^	F^−^	HCO_3_^−^
pH	1	0.389	0.542	− 0.21	− 0.194	− 0.47	**0.77**	− 0.303	− 0.556	0.484	0.433	0.64	0.508
TDS	0.389	1	**0.718**	0.349	0.459	0.202	**0.73**	− 0.124	0.028	**0.973**	0.527	0.65	0.642
TA	0.542	**0.718**	1	− 0.101	− 0.146	− 0.167	0.783	− 0.188	− 0.182	0.659	0.55	0.60	**0.977**
TH	− 0.21	0.349	− 0.1	1	0.944	**0.884**	− 0.004	0.233	0.59	0.223	− 0.316	0.047	− 0.248
Ca^2+^	− 0.194	0.459	− 0.15	**0.944**	1	**0.802**	0.029	0.153	0.55	0.385	− 0.255	0.097	− 0.276
Mg^2+^	− 0.47	0.202	− 0.17	**0.884**	**0.802**	1	− 0.31	0.53	0.546	0.037	− 0.417	− 0.3	− 0.283
Na^+^	**0.778**	**0.73**	**0.78**	− 0.004	0.029	− 0.313	1	− 0.489	− 0.126	**0.761**	0.666	**0.83**	**0.759**
K^+^	− 0.303	− 0.124	− 0.19	0.233	0.153	0.53	− 0.49	1	0.007	− 0.213	− 0.316	− 0.31	− 0.266
SO_4_^2−^	− 0.556	0.028	− 0.18	0.59	0.55	0.546	− 0.12	0.007	1	− 0.102	− 0.382	− 0.18	− 0.178
Cl^−^	0.484	0.973	0.659	0.223	0.385	0.037	**0.76**	− 0.213	− 0.102	1	0.593	**0.70**	0.597
NO_3_^−^	0.433	0.527	0.55	− 0.316	− 0.255	− 0.417	0.66	− 0.316	− 0.382	0.593	1	0.63	0.568
F^−^	0.642	0.654	0.607	0.047	0.097	− 0.3	**0.83**	− 0.312	− 0.184	**0.705**	0.635	1	0.516
HCO_3_^−^	0.508	0.642	**0.977**	− 0.248	− 0.276	− 0.283	**0.76**	− 0.266	− 0.178	0.597	0.568	0.516	1

Significant values are in bold.

A strong correlation coefficient between different water quality parameters is seen at those places where the climatic conditions are humid like Assam (India). The fluoride content in these water samples are increasing in these arid and semi-arid climate regions because of the slow rate of water percolation through the ground^43,55^. Increase in concentration of OH^−^, HCO^3−^, and CO_3_^2−^ results in increase in alkalinity of water sample. Various studies carried out in different regions of the world show that desorption of as ion and F^−^ from metallic oxide surface causes higher pH of the sampled water which is also confirmed by various experimental studies^30,43,65^. Table 7 provides a result of the impact of these variables on release of two pollutants separately and on the conductive environment for co-occurrence.

**Table 7:** Conducive environmental conditions for occurrence fluoride^58–63,66^.

Sources	Conditions contributing to fluoride release
Geogenic origin	From fluoride minerals like cryolite, adsorbed fluoride on clay minerals
Anthropogenic (Manmade)	Extraction of mineral through activities of mining, phosphate fertilizer effluents
pH	High concentration of fluoride in groundwater is due to phenomena of leaching which is favored by high pH of water
Organic matter	Increase the content of fluoride in potable water with a higher organic matter content
Redox conditions	High fluoride concentration affects the quality of environment
Concentration of ions	Few cations (Na, K, Ca, etc.) and anions (Bicarbonates) are found with high concentration of fluoride in water
Climatic conditions	Semi-arid to arid

### WQI and spatial distribution

In the area under investigation, it has been observed that water quality index ranges from 105 to 185, delineated as per the Table 8. Therefore, ‘special treatment’ is needed in the study area, to qualify in ‘fit water’ category. It was found that the ions like F^−^, Cl^−^, Na^+^ and alkalinity were above the permissible limit, resulting high total dissolved solids (TDS) value, which might be the cause of geogenic activities.

**Table 8:** Water quality index (WQI) values at Achhnera block, Agra region, North India.

S. no	Name of village	Water quality indexing (WQI)	Water quality
1	Arsena	158.115	Unfit for drinking
2	Biara	164.838	Unfit for drinking
3	Janutha	140.294	Unfit for drinking
4	Kukathala	122.349	Unfit for drinking
5	Loh Karera	162.857	Unfit for drinking
6	Raibha	185.866	Unfit for drinking
7	Mahuar	99.323	Poor quality
8	Runkata	152.101	Unfit for drinking
9	Sehta	124.867	Unfit for drinking
10	Achhnera	131.918	Unfit for drinking

**Figure 7:**
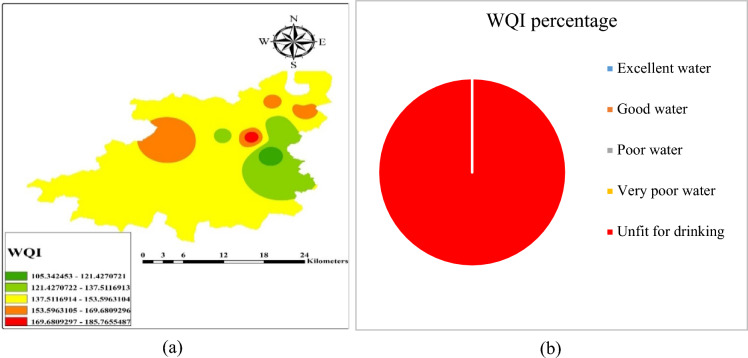
(**a**) Spatial distribution of WQI in the study area and (**b**) graphical data representation of WQI classifications of Achhnera block, Agra region, Uttar Pradesh, North India.

Through (Inverse Distance Weighted) IDW methods, the spatial distribution of factor scores was interpolated (Fig. 7). The graphical presentation of the WQI of the Achhnera block is illustrated in Fig. 7a. Based on the measurement of physiochemical aspects of different samples, taken from different locations and the WQI range of the sampled region is shown in the pie chart (Fig. 7b). From the analysis of the different water quality parameters, it was found that all the calculated values surpass the permissible limits suggested by WHO & BIS^38,39^, which results in a high level of TDS values. It is concluded from the experimental results that the high percentage of fluoride in the samples of Achhnera block, Agra district may be due to its geological conditions and the water of this region is unfit for drinking and cannot be used for various other purposes.

### Comparative study of the rural and urban areas using water quality parameters

**Figure 8:**
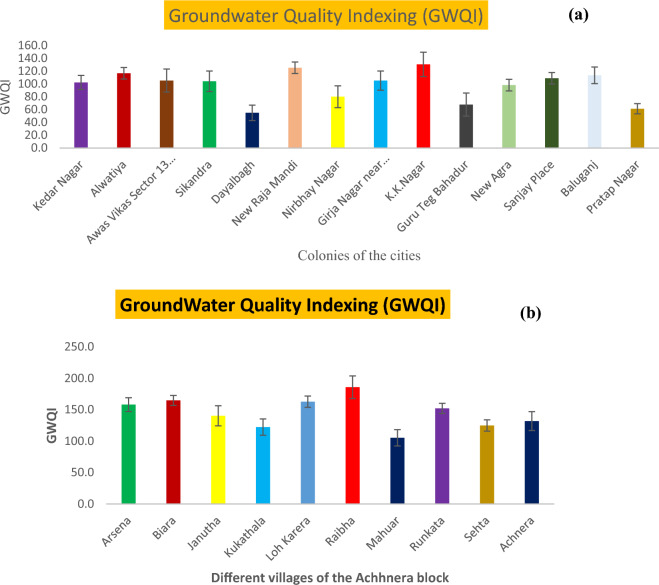
Bar chart of WQI comparative study with standard deviation of urban (**a**) and rural (**b**) areas of Agra region, Uttar Pradesh, Northern India.

It showed the comparative study done on the extent of pollution in drinking water between urban areas and the rural areas of Agra district of northern India. Study on GWQI of urban areas was previously carried out by Ali et al.^56^ and current study is based on rural areas of Agra district of northern India (Fig. 8). Twelve important water quality parameters were compared in groundwater quality analysis (GWQI). The GWQI of urban area were ranged from 50.01 to 130.62, which reveals that more than half of the urban region was found in the category unfit for drinking (64%), nearly one fourth of the region lies in the poor category range (21.42%) and the remaining region lies in the very poor category range (14.28%). Figure 8a, showed that no samples lie in the category of good or excellent. It was inferred that the large value of water quality index at urban regions was due to the geogenic as well as with some anthropogenic source (outlet of fertilizer industry).

Present study in, Achhnera block, shows that the WQI ranges from 185 to 105, delineated in Fig. 8b. The analysis of WQI revealed that the sampled area lies in the unfit category for the drinking purpose. Therefore, it is advised that the drinking water should be treated before making it suitable for drinking in Achhnera block of Agra region, Northern India. It can be concluded from the comparative analysis that potable water of the rural areas is comparatively more polluted than that of the urban areas, which may be due to geogenic as well as anthropogenic activities (use of fluoride laden fertilizer in the field, leeches into the groundwater)^13–15^.

## Conclusions

The present study has been performed to evaluate the groundwater quality for Achhnera block in the city of Taj, Agra, India, where groundwater is an important water resource. Therefore, this study was designed to the applicability of GWQI, and the PCA to mark out the major geochemical solutes responsible for origin and release of geochemical solutes into the groundwater. This study confirms that, majority of the groundwater samples in the study areas were mostly alkaline in nature. Elevated values of electrical conductivity, total dissolved solid, total hardness, fluoride and chloride in groundwater samples were mainly due to rock water interaction and high rate of evaporation. The results conclude that the water quality index belongs to unfit category for potable use in the study area, hence, almost all sampling tube-wells of the study area fell in the category of unsuitable for drinking purpose. Further, hydrochemistry of groundwater confirms that, most of the collected groundwater samples in the study area were comparatively saltier than freshwater. The Piper diagram concludes that, the major hydro chemical facies found in groundwater were sodium bicarbonate type or calcium chloride type. Finally, the PCA shows the pH, Na^+^, Ca^2+^ and fluoride with high loading, suggests geogenic source of fluoride contamination. Therefore, it is recommended that the water of Achhnera block of Agra region Northern India, should be treated properly before use as potable water. It can be concluded from the comparative analysis that regions of the rural areas are comparatively more polluted than that of urban areas, which may be due to geogenic as well as anthropogenic activities (use of fluoride laden fertilizer in the field, leeches into the groundwater). Complete distribution of physico-chemical characteristics of water is shown in this study which can be used as a tool to improve the water quality for drinking purposes.

## Data Availability

The datasets generated and analyzed during the current study were available from the corresponding author on reasonable request.
